# Sweet but Bitter: Focus on Fructose Impact on Brain Function in Rodent Models

**DOI:** 10.3390/nu13010001

**Published:** 2020-12-22

**Authors:** Maria Stefania Spagnuolo, Susanna Iossa, Luisa Cigliano

**Affiliations:** 1 Department of Bio-Agrofood Science, Institute for Animal Production System in Mediterranean Environment, National Research Council, I-80147 Naples, Italy; mariastefania.spagnuolo@cnr.it; 2 Department of Biology, University “Federico II”, I-80134 Naples, Italy

**Keywords:** fructose diet, GLUT5, neuroinflammation, brain oxidative stress, brain mitochondria, brain insulin resistance, cognitive impairment

## Abstract

Fructose consumption has drastically increased during the last decades due to the extensive commercial use of high-fructose corn syrup as a sweetener for beverages, snacks and baked goods. Fructose overconsumption is known to induce obesity, dyslipidemia, insulin resistance and inflammation, and its metabolism is considered partially responsible for its role in several metabolic diseases. Indeed, the primary metabolites and by-products of gut and hepatic fructolysis may impair the functions of extrahepatic tissues and organs. However, fructose itself causes an adenosine triphosphate (ATP) depletion that triggers inflammation and oxidative stress. Many studies have dealt with the effects of this sugar on various organs, while the impact of fructose on brain function is, to date, less explored, despite the relevance of this issue. Notably, fructose transporters and fructose metabolizing enzymes are present in brain cells. In addition, it has emerged that fructose consumption, even in the short term, can adversely influence brain health by promoting neuroinflammation, brain mitochondrial dysfunction and oxidative stress, as well as insulin resistance. Fructose influence on synaptic plasticity and cognition, with a major impact on critical regions for learning and memory, was also reported. In this review, we discuss emerging data about fructose effects on brain health in rodent models, with special reference to the regulation of food intake, inflammation, mitochondrial function and oxidative stress, insulin signaling and cognitive function.

## 1. Introduction

In the last forty years, the severe modifications of human diet composition have introduced new potential risk factors for the development of metabolic diseases. In particular, the extensive commercial use of high-fructose corn syrup (HFCS) as sweetener for beverages, tea, coffee, snacks and baked goods has caused a strong rise in the fructose content of the human diet [1,2]. Evidence from epidemiological studies has suggested that fructose represents a potential contributor to the worldwide rise in being overweight and in body weight gain [1,2,3]. Further, both clinical investigations and animal studies demonstrated that a high fructose diet is associated with the onset of dyslipidemia, insulin resistance and related metabolic diseases [4,5,6,7]. It was proposed that the unique metabolism of fructose is partially responsible for the role played by this sugar in the etiology of several metabolic diseases [8,9]. The primary metabolites and by-products of fructolysis in liver are glucose, lactate, free fatty acids, very low-density lipoproteins, uric acid and methylglyoxal, which can impair the function of extrahepatic tissues and organs. However, fructose itself causes an adenosine triphosphate (ATP) depletion that triggers an inflammatory response and oxidative stress. For an extensive review on the effect of fructose on the liver, adipose tissue, pancreas islet, skeletal muscle, kidney, heart and small intestine, readers are directed to References [10,11,12,13,14].

**Figure 1:**
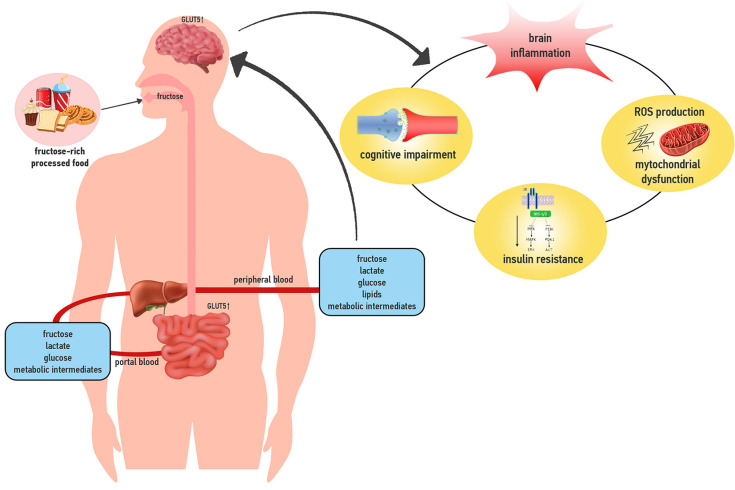
Fructose is cleared by the small intestine, which converts it into different metabolic intermediates. High fructose doses overwhelm the intestinal capacity for fructose metabolism, and a part of fructose spills over to the liver, which can metabolize it. Gut- and liver-derived metabolic intermediates or fructose itself can reach the brain, exerting effects on regulation of food intake, brain inflammation, mitochondrial function and oxidative stress, insulin signaling and cognitive function. Down arrow indicates downregulated and up arrow indicates upregulated cellular pathways by fructose intake. GLUT5 = glucose transported 5.

This review will focus on emerging data about fructose impact on the brain, with special reference to the mechanisms and biochemical pathways related to regulation of food intake, inflammation, mitochondrial function and oxidative stress, insulin signaling and cognitive function (Figure 1).

## 2. Fructose Effects on the Regulation of Food Intake

Central administration of fructose stimulates feeding in rodents [15,16], and it was recently reported that the chronic consumption of a fructose-containing diet induces a strong preference and motivation for this diet [17], likely due to fructose palatability and sweeter taste perception. Therefore, a fructose-induced modification in the function of the brain reward circuitry was hypothesized [17]. The hypothalamus is the appetite center and the major energy status sensor, which acts through neuropeptides secretion that occurs via the regulation of adenosine monophosphate (AMP)-activated protein kinase (AMPK) signaling and malonyl-coenzyme A (CoA) concentration. Fructose-induced health disturbances are different from those provoked by glucose or sucrose, likely because the initial steps of fructose metabolism differ from those of glucose not only in the liver but also in the central nervous system (CNS). In fact, fructose bypasses the rate-limiting regulatory step of glycolysis and is therefore metabolized far more rapidly than glucose. This leads in the hypothalamus to a rapid depletion of ATP [16], accompanied by an increase in AMP level, the activation of AMPK, the inactivation of acetyl-CoA carboxylase and a decrease of malonyl-CoA concentration, thus increasing food intake and risk of obesity [16,18]. Fructose also influenced the release of, or the response to, hormones regulating appetite and food intake. Long-term (six months) fructose feeding in the rat was reported to induce leptin resistance without affecting circulating hormone levels, and this effect was ascribed to a defective transport of leptin through the blood brain barrier (BBB) due to increased circulating triglycerides [19]. Conversely, short-term fructose feeding was demonstrated to induce a lower rise in comparison to glucose in the peripheral concentrations of hormones that increase satiety and reduce hedonic related feeding, such as insulin, glucagon-like polypeptide-1 and leptin [20,21,22]. Further, a short-term high fructose diet increased the hunger hormone ghrelin, depressed the satiety signal peptide YY3-36 and down-regulated hypothalamic neuropeptide Y (NPY) mRNA, thus producing a cooperative effect in the induction of appetite and food intake [23]. In contrast to the short-term effects, fructose treatment for nine weeks upregulated NPY mRNA and elicited leptin resistance in the hypothalamus [24].

Collectively, these studies on rodent models have evidenced that fructose and glucose differently impact endocrine systems and cerebral pathways regulating appetite and feeding behavior.

## 3. Fructose and Neuroinflammation

Although various studies have shown that diet-induced obesity is associated with increased brain oxidative stress [25,26,27,28] and neuroinflammation [29,30,31,32], the majority of these results has been obtained using high fat and/or high fat/high sugar diets.

Fructose-induced inflammatory responses in peripheral tissues, especially in the liver, muscles and kidneys have been documented [33,34,35,36,37,38], and, in the last years, attention has also been focused on the ability of this sugar to induce neuroinflammation [32,39,40]. Fructose consumption was shown to induce psychological stress through an inflammatory mechanism [41]. Four weeks of fructose feeding induced increased expression of the proinflammatory mediator genes Interleukin (IL)-1beta, IL-6 and Tumor necrosis factor (TNF)-alpha, together with Toll-like receptor 4 (TLR4), myeloid differentiation factor 88 and nuclear factor kappa-light-chain-enhancer of activated B cells (NF-kB), in the hypothalami of rats [42]. Fructose feeding was shown to be associated with increased levels of histone deacetylases 3 (HDAC3), thus suggesting that HDAC3 represents a crucial component in the network linking fructose to neuroinflammation in metabolic syndrome, through the activation of TLR4/NF-kB pathway [42].

The pro-inflammatory effect of fructose was also evidenced in other brain regions. Excess consumption of HFCS exerted detrimental effects on hippocampal function and caused neuroinflammation in the CNS during adolescence [43]. Of note, fructose feeding induced hippocampal microglia activation through the stimulation of TLR4/NF-kB signaling, resulting in the reduction of neurogenesis in the dentate gyrus of mice [44]. The chemokine fractalkine and its receptor CX3C Chemokine Receptor 1 were reported to participate in fructose-induced neuro-inflammation via the activation of TLR4/NF-kB signaling [44]. Long-term fructose-drinking (16 weeks) caused neuroinflammation associated with impaired insulin signaling, oxidative stress, reduced activity of the cholinergic system and cognitive impairment in both the hippocampi and the cerebral cortices of rats [45]. Djordjevic et al. [46] reported that long-term consumption of 10% fructose solution induced an increase in IL6, with no change in NF-kB activation, while, in rats drinking 60% fructose, significant NF-kB elevation was found, although all of the analyzed cytokines were unchanged. The explanation of this result might be found in the fact that hippocampal glucocorticoid signaling was higher in rats drinking 60% fructose so that the mutually opposing actions of glucocorticoid receptors and NF-kB resulted in the unchanged expression of proinflammatory genes. Further, the consumption of a high fructose diet (55% of energy from fructose) throughout adolescence and during adulthood induced the expression of genes of the complement pathways in the rat hypothalamus and hippocampus [47].

It is of note that recent experimental evidence has demonstrated that even a short period of treatment with fructose can have a detrimental impact on brain neuroinflammation. In fact, a very short-term (seven days) fructose rich diet was shown to induce morphologic, structural and functional modifications in a rat hippocampus, associated with an increase in reactive astrocytes and microglial activation [17]. In contrast, Jimenez-Maldonado [48] advocated that a short period (just one week) of fructose can disturb neuronal integrity and brain plasticity without inducing significant astroglial or microglial activation.

Although young and adults differ largely in their metabolic and physiological profiles, most of the mentioned studies investigated fructose-induced brain disturbances in adults. In this regard, our research group showed that a short-term consumption of fructose (two weeks) produces an early increase in specific markers of inflammation (TNF-alpha and glial fibrillary acidic protein) in the hippocampi of both young and adult rats [39]. This diet also induced, in the frontal cortices of both age groups, the activation of autophagy, as well as the decrease of both peroxisome proliferator-activated receptors, (PPAR)-alpha and PPAR-gamma, which are known to possess neuroprotective, anti-inflammatory and anti-oxidant properties [49].

## 4. Fructose, Brain Mitochondrial Function and Oxidative Stress

Although the brain represents only 2% of the body weight, it receives 15% of cardiac output and accounts for 20% of total body oxygen consumption. Mitochondria are the subcellular organelles that generate the energy needed for normal cellular function. In addition, they are involved in the mechanisms that initiate a programmed cell death. In particular, mitochondria are essential for neuronal function since these cells possess a limited glycolytic capacity and are highly dependent on aerobic oxidative phosphorylation for their energetic needs [50].

Oxidative phosphorylation is a major source of endogenous reactive oxygen species (ROS), which are byproducts of normal cellular respiration [51]. In particular, the dysfunction of the mitochondrial energy metabolism leads to reduced ATP production, impaired calcium buffering and the generation of reactive oxygen species (ROS), which are increasingly recognized as playing an important role in neurodegenerative diseases because of their ability to cause oxidative stress and consequently damage cellular contents [52]. For these reasons, it is not surprising that any impairment in mitochondrial activity places neurons at a high risk for dysfunction and/or death, thus indicating that mitochondrial alteration and oxidative damage may play a key role in the pathogenesis of neurodegenerative diseases [50].

Evidence is accumulating on the challenges imposed by high fructose consumption on brain mitochondrial function and potential neurological disorders, in agreement with the association between metabolic disease and disturbances in cognition and emotional health and reduced quality of life. A significant decrease in major markers of mitochondrial function has been detected in the brain after high fructose feeding in both short- and long-term experiments. Jiménez-Maldonado [48] evidenced, even after one week of fructose feeding, a reduction in peroxisome proliferator-activated receptor gamma coactivator-1 alpha (PGC1-α) and Cytochrome c oxidase subunit II, indicative of mitochondrial dysfunction in the hippocampus. Similarly, we have found that mitochondrial function in the hippocampus, together with PGC1-α content, was significantly decreased in fructose-treated adult rats after only two weeks [39].

Agrawal et al. [53] showed that fructose consumption, after eight weeks, disrupts hippocampal energy homeostasis, as evidenced by a decline in mitochondrial bioenergetics (oxygen consumption rate and cytochrome C oxidase activity), coupled with increased plasma membrane lipid peroxidation. In addition, a widespread reactive gliosis and altered mitochondrial respiratory complexes activity have been evidenced in the hippocampi of rats fed a 60% fructose diet for 12 weeks, paralleled by an oxidative stress increase due to the impaired activity of nuclear factor (erythroid derived 2)-like 2 (Nrf2) signaling [54].

In another study on the impact of acute fructose administration, rats were subjected to subcutaneous injection of fructose (5 μmol/g of body weight), and increased activity of malate dehydrogenase (MDH), without changes in the activity of the other enzymes of the Krebs cycle, was observed in the cerebral cortex 24 h after fructose injection [55]. The enhancement of the MDH activity was suggested to lead to an increase of the nicotinamide adenine dinucleotide (NAD)H/NAD+ ratio, negatively impacting bioenergetics, and it was thus considered a risk factor for cognitive function and neuronal plasticity [55].

An interesting set of data evidenced that even maternal fructose intake during gestation and lactation impaired brain mitochondrial function of the offspring. In fact, Mortensen et al. [56] evaluated mitochondrial function in the brains of aging (15 months) male offspring of Fischer F344 rat dams fed a high-fructose diet (50% energy from fructose) during gestation and lactation, and found a significant increase in state 3 respiration, as well as a significant decrease in the P/O ratio. This finding evidenced that maternal high-fructose diet during gestation and lactation has long-term effects (fetal programming) on brain mitochondrial function in aging rats. Very recently, Wu et al. [57] reported that maternal fructose intake during gestation and lactation impaired astrocytic glycolysis and mitochondrial oxidative phosphorylation, while also suppressing the mitochondrial DNA copy number and the expression of mitochondrial transcription factor A (TFAM) in mitochondria. In addition, Yamada et al. [58] showed that maternal high fructose consumption attenuated the mitochondrial O_2_ consumption rate, stimulated lipid hydroperoxide production and reduced *Tfam* mRNA levels after maternal exposure to fructose in the hippocampi of offspring.

Recently, a study on both male and female rats investigated the effect of a long term (12 weeks) high fructose diet on synaptic mitochondrial respiration, which was considered to be the potential intersection between diet-induced energetic imbalances and behavioral modification [59]. Indeed, male rats showed a decrease in synaptosomal oxygen consumption rate, while, in treated female rats, mitochondrial respiration increased and this resilience was ascribed to a neuroprotective effect of estrogens. Dietary treatment did not affect social interaction in either sex but induced, only in females, an increased depressive-like behavior, while in male rats it decreased anxiety-like behavior.

Although various studies evidenced the alteration of redox homeostasis induced by excessive fructose consumption at the systemic level [33,60,61], only in the last decade the effects of this sugar on redox balance in the CNS have been explored. Interestingly, an increase in oxidative stress was found both after short-term and long-term fructose intake. The acute effect of fructose on oxidative imbalance was demonstrated on 30-day-old male rats that received a single subcutaneous injection of fructose (5 μmol sugar per gram of body weight). In fact, this treatment induced in the cerebral cortex the oxidation of lipids and proteins and changes in the catalase and superoxide dismutase activity [62]. In addition, our research group demonstrated that a two-week fructose diet was associated with hippocampal oxidative stress, as increased levels of lipid and protein oxidation was detected in this brain area both in young and adult rats [39]. The imbalance of redox homeostasis was also found in the frontal cortex of both fructose-fed young and adult rats, with lower amounts of Nrf2, lower activity of Glucose 6-phosphate dehydrogenase and Glutathione reductase and a lower Glutathione (GSH)/Oxidized Glutathione (GSSG) ratio [49]. Since autophagy is usually activated as a protective mechanism in response to oxidative stress, we also showed for the first time that a short-term fructose-rich diet is associated with the activation of autophagy, as increased levels of beclin 1, microtubule-associated protein 1A/1B-light chain 3 and phospho sequestosome 1 were detected in frontal cortices of rats of different ages [49].

As for long-term fructose effects, rats receiving fructose supplementation (10% *w/v* in drinking water) for 16 weeks showed significantly higher levels of ROS, lipid peroxides, carbonyls and lower levels of GSH compared to control rats in both the hippocampus and cerebral cortex, associated with lower activities of superoxide dismutase, catalase and glutathione peroxidase in both brain areas [45].

In a further investigation, Sanguesa et al. [63] showed that fructose supplementation (10% *w/v* in drinking water) for seven months (28 weeks) reduced the expression of antioxidant enzymes and altered the number of proteins involved in mitochondrial fusion/fission in the frontal cortex [63]. Interestingly, by using the degu, a long-lived animal model, the first long-term study that replicates human fructose consumption was recently performed to understand the mechanisms by which metabolic disorders could affect cognitive abilities. As well as systemic metabolic alterations, excessive fructose intake also induced several neuropathological events, such as oxidative stress processes, causing hippocampal lipid peroxidation and protein nitrosylation, together with synaptic protein loss. These changes were associated with alterations in synaptic plasticity and transmitter release in these cognitively impaired animals [64]. This evidence was corroborated in a further study on Syrian hamsters, small rodents that have many features that resemble human physiology. In this animal model, fructose intake (60% for ten weeks) led to alterations of the unfolded protein response and macroautophagic machinery in the brain, favoring the accumulation of aggregates of β-amyloid 1-42 (Aβ42), tau-p-S199 and tau-p-S404, well known markers of neurodegeneration [65].

Finally, fructose has been reported to be a more potent glycating agent than other sugars [7,66,67]. In agreement, a high fructose intake was shown to induce the production of advanced glycation end products (AGEs), specifically of carboxy methyllysine, which involves impairment of the activity of the AGEs-detoxifying enzyme glyoxalase-1 in the hippocampi of C57Bl/6 mice [54]. These findings are of relevance since different studies indicated the involvement of AGEs in brain dysfunction and neurodegenerative diseases [68,69,70].

## 5. Fructose and Insulin Signaling

Insulin resistance impairs brain function, and a large body of evidence suggests a link between impaired insulin function and the risk of developing Alzheimer’s Disease (AD) and other neurodegenerative diseases [71,72,73,74,75,76]. Indeed, the term “type 3 diabetes” has been used to describe AD, as it represents a form of diabetes that selectively involves the brain and has molecular and biochemical features overlapping both type 1 and type 2 diabetes mellitus (T2DM) [77]. Further, in early AD progression, an insulin resistant state was observed [78,79], and several epidemiological studies have identified T2DM as a risk factor for developing AD [80,81]. Insulin-dependent pathways have been suggested as crucial for longevity [82] and brain aging. Therefore, a decrease of CNS sensitivity to insulin has been proposed as the potential link between metabolic and cognitive dysfunctions [83,84], and the multiple roles of insulin in the brain, as well as the effects of brain insulin resistance, have been extensively reviewed by Kullman [85]. In 2005, an investigation on fructose-fed hamsters revealed, for the first time, that fructose feeding was associated with a reduction in the tyrosine phosphorylation status of the insulin receptor and insulin receptor substrate 1 (IRS-1), as well as in the serine/threonine protein kinase B (Akt) activity in the brain. The authors also demonstrated a significant increase in the levels of the protein tyrosine phosphatase 1B, which was suggested to contribute to neural insulin resistance by dephosphorylating upstream components of the insulin cascade [86].

More recently, it was reported that rats receiving 10% fructose in drinking water for 10 weeks developed a hypothalamic insulin signaling defect, detected by a reduction in insulin receptors and Akt phosphorylation [87]. Increased pAMPK and phospho-acetyl-CoA carboxylase protein levels and overexpression of thioredoxin-interacting protein (TXNIP), associated with the dysfunction of the glutamine-glutamate cycle, was also found in the hypothalami of fructose fed rats. In addition, the activation of NF-kB and NOD-like receptor 3 inflammasome pathways as a consequence of AMPK/TXNIP dysregulation was found. The authors proposed that the hypothalamic insulin signaling defect may be produced by fructose itself through the involvement of protein kinases AMPK and TXNIP, which are fructose-sensitive nutrient sensors. In another study, male Sprague-Dawley rats fed for eight months with a high fructose diet showed an attenuation of the insulin signaling pathway in the hippocampus, together with hippocampal-dependent memory impairments [88]. It was hypothesized that hippocampal insulin resistance and memory impairment might represent the direct consequence of peripheral insulin resistance induced by a high-fructose diet. An interesting investigation [46] reported that male Wistar rats (21 days) exposed to high fructose by drinking a 10% fructose solution for nine weeks showed impaired peripheral insulin sensitivity, elevated levels of hippocampal inhibitory Ser307 phosphorylation of IRS-1, increased hippocampal levels of IL-6 mRNA and decreased levels of TNF-alpha and IL-1β mRNAs. Conversely, in rats drinking a 60% fructose solution, hippocampal insulin resistance was not observed, and cytokines remained unchanged. These differences were ascribed to the increase in glucocorticoid signaling in the rats drinking 60% fructose. The main finding from this investigation was that disturbances in peripheral and hippocampal insulin sensitivity are concomitant events not necessarily related to adiposity and that the response to chronic fructose loading strongly depends on the amount of fructose [46].

More recently, it was demonstrated that a diet containing 60% fructose induces, in adult male Wistar rats after 12 weeks, peripheral and hippocampal insulin resistance [89]. In this study, a diet-induced decrease in brain derived neurotrophic factor (BDNF) was also evidenced, and the authors supposed a negative control exerted by insulin resistance on BDNF expression. In addition, the observed postsynaptic impairment of the preexisted neuronal circuit was ascribed to the decreased expression of BDNF protein. Further, the condition of insulin resistance was proposed to be involved in the suppression of adult neurogenesis in the dentate gyrus of the hippocampus found in high fructose fed rats [89]. In another study on female rats, it was demonstrated that long term exposure (nine weeks) to a high fructose diet, combined with chronic stress, elicited an impairment in insulin signaling while activating the glucocorticoid pathway in the hypothalamus, leading to the hypothesis that increased glucocorticoid signaling could disturb insulin signaling. It was also suggested that reduced insulin signaling might contribute to an increase in oxidative damage in this compartment [90]. Interestingly, short-term fructose feeding (two weeks) was shown to be associated with a decreased amount of hippocampal IRS and Akt phosphorylation in young rats [39], thus suggesting an impairment of insulin signaling in agreement with the systemic insulin resistance found at the whole-body level in these rats. In the same experimental model, adult rats showed decreased IRS but increased Akt phosphorylation, together with extracellular signal-regulated kinase (Erk) activation, thus suggesting that adult rats are more sensitive to the detrimental effects of fructose, as an accumulation of active phosphorylated Akt was described in models of neurodegeneration.

A recent study [57] investigated the effect of a maternal high fructose diet during gestation and lactation on the astrocytic glucose metabolism of offspring in order to identify a possible molecular link between maternal overconsumption of fructose and the impairment of offspring hippocampal function. The authors used hippocampal primary cultures of female infants and observed an astrocytic deficiency of glycolysis, the downregulation of GLUT1 transporter and the upregulation of insulin signaling [57]. Hence, they proposed that the enhancement of astrocytic insulin signaling represents a compensatory mechanism in response to the lower glucose uptake and metabolism [57]. However, the effective link between maternal diet and the alteration of metabolism, learning and memory in offspring remains still largely unknown.

## 6. Fructose, Cognitive Function, Aging and Neurodegenerative Diseases

One of the first research studies evidencing the impact of fructose on learning and memory was performed on male Sprague Dawley rats fed a long-term (eight months), high-fat, high-glucose diet supplemented with 20% HFCS in the drinking water. The authors revealed that this dietary treatment, mimicking the Western diet, was associated with a reduction in the hippocampal BDNF level and with the impairment of hippocampus-dependent learning and memory, synaptic plasticity and dendritic spine density [91]. As the treated rats showed a serum profile resembling diabetes, the authors supposed that peripheral insulin resistance, hyperlipidaemia and endocrine perturbations induced by excessive caloric intake might alter hippocampal structure and function.

Interestingly, some studies also explored the impact of dietary fructose at different life stages. It was reported that ad libitum consumption of an 11% HFCS-55 solution for 30 days in young and adolescent, but not adult rats, impaired hippocampal-dependent spatial learning and memory retention [43]. Similarly, a high-fructose diet during adolescence elicited increased anxiety-like and depressive-like behavior in adulthood, while these responses did not occur in rats fed a high fructose diet during adulthood only [92]. Differently from these results, a study on Sprague Dawley rats revealed that high fructose diet (55% kcal from fructose) feeding from adolescence until adulthood, although disrupting animal metabolism, did not affect anxiety-like or depressive-like behavior, nor did it modify the intrinsic excitability of basolateral amygdala principal neurons [93]. These findings suggest that the effect of a fructose rich diet on affective behavior may be tightly dependent on strain and age.

Short-term fructose feeding (10% *w/v*, fructose solution for five weeks) was shown to alter the sleep-wake cycle in young rats, eliciting wake-promoting effects and reducing the time spent in non-rapid eye movement sleep [94]. This effect was considered a consequence of the fructose-induced activation of orexinergic neurons in the hypothalamus and of dopaminergic neurons in midbrain regions [94].

Several studies suggest that the effects of fructose on synaptic plasticity and cognitive function might be a consequence of inflammation and the impairment of brain insulin signaling induced by this sugar. Male Syrian hamsters fed a 60% fructose diet for six weeks developed hippocampal insulin resistance associated with a significant attenuation of insulin-induced long-term depression [86]. Since the hippocampus is integral to many forms of learning and memory [95] and neural insulin signaling facilitates hippocampal-dependent memory [96], the authors hypothesized that a high fructose diet may contribute to cognitive impairment by impairing the brain insulin signaling pathway.

The effect of fructose was later confirmed by Ross et al. [97], who demonstrated an impairment of hippocampal-dependent memory in male Sprague Dawley rats fed a diet containing 60% fructose for 19 weeks [97]. They found that long-term feeding on a high fructose diet impairs hippocampal-dependent spatial water maze retention performance, specifically affecting long-term storage and/or retrieval processes. Conversely, the diet did not impair acquisition performance during training. The authors also reported a significant correlation between fructose-induced retention deficits and fructose-induced increases in liver mass and plasma triglycerides (TG) concentrations.

Consumption of ad libitum drinking water containing 23% fructose over a short period (four weeks) was found to be associated with a reduction of neurogenesis and an increase of apoptosis in the dentate gyrus of the hippocampus [98]. The increase of apoptosis was ascribed to the observed increase in circulating TNF-alpha, while the reduced transport of ghrelin and leptin across the BBB was responsible for reduced hippocampal neurogenesis [98].

The attenuation of the insulin-signaling pathway in the hippocampus and the impairment of learning and memory, as assessed with a Morris water maze test, were demonstrated by Wu et al. [88], who analyzed the effect of fructose beverages by feeding male Sprague Dawley rats for eight months with a 10% fructose solution. As insulin resistance was found to be positively correlated with mean escape latency, they suggested that fructose-induced peripheral insulin resistance may contribute to hippocampal-dependent cognitive function, either because chronic hyperinsulinemia might determine a reduced insulin transport into the brain or because a fructose diet induces an increase in plasma TG, which penetrate the BBB and promote hippocampal-dependent memory deficits [99]. An investigation performed with two-month-old C57BL/6 male mice demonstrated that the administration of normal food and water supplemented with 15% fructose for eight weeks induced disruption of hippocampal neuronal excitability, with consequences for brain function and plasticity [100]. As a matter of the fact, the treated mice lost their ability to sustain hippocampal long-term potentiation and long-term depression and also showed a significant decrease in the density and size of active zones at synapses, as well as decreased expression of the glutamate receptor subunits NMDAε1 and GluR2, thus indicating a deregulation of hippocampal synaptic transmission and plasticity. Further, a significant reduction in adult hippocampal neurogenesis and an impairment in spatial memory performance were also reported [100].

In a study with Sprague Dawley female rats exposed to 10% fructose in drinking water for seven months, it was evidenced that fructose impairs nonspatial memory and that the memory deficit depends mostly on the cortex, where the insulin-signaling impairment was more intense compared to the hippocampus [63]. Long-term fructose intake impaired cognitive performance evaluated with the novel object recognition test but did not affect learning and memory as assessed by the Morris water maze test. Further, a significant reduction in the protein level of BDNF was found in the hippocampus, although the levels of other proteins related to synaptic plasticity, such as synapsin 1, growth-associated protein 43, synaptophysin and postsynaptic density protein 95 (PSD-95), were not affected by treatment [63].

Ad libitum access to a solution of 60% or 10% fructose for nine weeks revealed that the level of Polysialylated neural cell adhesion molecule (PSA-NCAM), a marker of synaptic plasticity, was significantly reduced only in the hippocampi of rats drinking 60% fructose [46]. The decrease of PSA-NCAM level was proposed to be a consequence of enhanced glucocorticoid signaling since increased levels of 11-beta-hydroxysteroid dehydrogenase type 1 and glucocorticoid receptors were also found in the hippocampi of fructose fed rats [46].

Similar results were obtained by measuring BDNF, another marker of synaptic plasticity that is critical for the positive regulation of hippocampal functions [101]. In a recent study on adult rats fed a high fructose diet (60% fructose for 12 weeks) [89], insulin resistance at both the peripheral and hippocampal levels, a reduction in the protein level of BDNF and an impairment at the postsynaptic level of the pre-existed neuronal circuit were found in the hippocampus, while the levels of Ca2^+^/calmodulin-dependent protein kinase II alpha and PSD-95 were also reduced. Similarly, we recently reported that a short-term (two weeks) fructose diet was associated with a decrease in the presynaptic proteins synaptotagmin I, synapsin I and synaptophysin I in young (30-days-old) and adult rats (90 days) [49]. Further, an impairment in BDNF signaling, in terms of a decrease in BDNF protein and/or receptor (TrkB) levels, was observed in the two groups, strongly supporting the idea that fructose-associated alteration of brain plasticity critically depends on neurotrophin action [49].

Collectively, these papers highlight the potential negative effects of fructose in the brain, with a series of studies showing cognitive deficits in spatial memory and learning. In contrast with these studies, Rendeiro et al. [102] showed no differences in hippocampal neurogenesis and cognitive/motor performance as measured by object recognition, fear conditioning and rotarod tasks. It should, however, be noted that the impact of a fructose diet was evaluated relative to an isocaloric glucose diet, rather than a control diet containing complex carbohydrates.

Notably, it has also been evidenced that a maternal high fructose diet has a profound impact on the offspring’s brain physiology. Impairment of spatial learning and memory performance, together with reduced levels of BDNF and the up-regulation of histone deacetylase 4 in the nuclear fractions of hippocampal neurons, was shown in adult female offspring rats from a mother fed with a 60% high fructose diet during pregnancy and lactation [103]. Similarly, a maternal high fructose diet (60%) altered anxiety-like behavior in young and adult rats [104]. Moreover, impairment in cognitive performance, likely due to reduced hippocampal neurogenesis, was detected in 60-day-old Sprague Dawley rats born from mothers that received 20% fructose water in addition to standard chow during gestation and lactation [105].

Another issue investigated in the last years is the possible role played by fructose consumption in the occurrence of pathophysiological characteristics of neurodegenerative diseases such as AD. Fructose administration (10% solution in drinking water for 16 weeks) induced, in rats, insulin resistance, brain oxidative stress and activation of inflammation and AGE-AGE receptor (RAGE) pathways, thus contributing to spatial memory impairment [106]. Similarly, it was reported that the consumption of 10% fructose solution in drinking water for 16 weeks was associated with Aβ42 deposition in both the cortex and hippocampus, the dysregulation of the Aβ metabolism, reflected in the increased expression of beta-secretase 1 and presenilin 1 and reduced levels of insulin degrading enzyme, a condition similar to that observed in AD patients [107].

A study on mice demonstrated that a long-term (20 weeks) 60% fructose diet induces the impairment of the detoxifying glyoxalase-1 enzyme and the hippocampal accumulation of carboxy methyllysine, which has been suggested to contribute to the development of many neurodegenerative diseases, such as AD, Parkinson’s disease and amyotrophic lateral sclerosis. In addition, treated mice showed activation of AGE/RAGE/NF-kB signaling, reactive gliosis, mitochondrial dysfunction and oxidative stress, which are typical events found in the major neurodegenerative diseases [54]. More recently, the molecular mechanism linking fructose overconsumption to brain cellular alterations was investigated in Syrian hamsters fed a high-fructose diet (60%) for 10 weeks [65]. The authors demonstrated that excessive consumption of fructose could favor the occurrence of primary events of AD, as they found increased amounts of Aβ42 and α-synuclein in the brains of fructose-fed hamsters, as well as increased phosphorylation of tau protein at S404, a critical site for microtubule assembly, and at S199, which is involved in the formation of neurofibrillary tangles [108,109]. They also found fructose-induced oxidative stress and dysfunction of quality control systems, such as the ubiquitin-proteasome system and autophagy. As a result, they proposed that fructose, altering the redox system and mechanisms involved in the detection and degradation of abnormal proteins, might give rise to early neurodegenerative alterations.

A very recent investigation tried to elucidate the effects of fructose-induced T2DM in a mouse model of AD [110]. Insulin resistance was induced by feeding 3xTg-AD mice with a diet containing 60% fructose, with free access to 10% fructose water for three months (from six to nine months of age). Fructose treatment did not exacerbate cognitive dysfunction in the mouse model of AD but induced microglial activation and brain insulin resistance. Further, the total amount of Aβ42 was not affected by dietary treatment, but a significant increase of toxic Aβ conformers and p-tau levels was observed. Therefore, the authors proposed that a fructose diet might facilitate toxic Aβ conformer production via the cross-seeding by p-tau oligomers.

Overall, it seems clear that the overconsumption of fructose can impair synaptic plasticity and cognition. Although the precise molecular mechanism has not been clarified yet, research in the field in the last decade strongly supports the hypothesis that peripheral hyperinsulinemia and insulin resistance associated with high fructose consumption are collectively considered to be key factors driving brain dysfunction in terms of gliosis, oxidative stress, synaptic plasticity impairment and the possible accumulation of toxic compounds, which can contribute to the pathogenesis of neurodegenerative diseases, such as AD.

## 7. Direct Effects of Fructose on the Brain

The systemic inflammation triggered by fructose can, in principle, induce neuroinflammation, brain oxidative stress and other metabolic perturbations through the import of inflammatory cytokines and/or other plasma metabolites into the CNS. An alternative hypothesis could consider the entry of fructose into the brain and its direct effect on the functional activities of neuronal and glial cells.

Dietary fructose is absorbed across the small intestine through glucose transporter 5 (GLUT5) [111]. The small intestine is crucial in the dietary fructose metabolism, converting it into glucose and other metabolites, thus protecting liver from fructose exposure [112]. In particular, the small intestine is able to clear about 90% of fructose, when physiological doses of sugar are ingested, while higher doses overwhelm the small intestine and pass to the liver, where they can cause toxicity [112]. In general, fructose is transported from the enterocytes into systemic circulation by GLUT2 and then metabolized via fructolysis in the liver and kidney [113,114]. The fructose not cleared by these two organs circulates in the blood and can, although in small amounts, reach the brain, as recently demonstrated with mice receiving an oral gavage of a mixture of ^13^C fructose: glucose, followed by mass spectrometry to track the fate of the glucose and fructose carbons in vivo [112]. For this to occur, fructose should pass the BBB. Interestingly, GLUT5 was found to be expressed in the BBB [115], microglia [116] and choroid plexus [117]. In addition, extracellular levels of fructose in the hypothalamic area of the rat were found to be similar to serum levels, and they responded to changes in serum levels [22]. This finding is of interest since it has been shown that fructose ingestion increases blood fructose concentration in both humans and rodents [118,119]. Once in the brain, fructose metabolism via the Fructose-1-Phosphate pathway can occur in cells expressing the GLUT gene, which is capable of constitutive fructose transport, the ketohexokinase gene and aldolase B or C genes. Bioinformatic analysis by Virtual Northern Blot revealed significant expression of genes necessary for fructose metabolism in the brain [120]. Actually, the ability of the brain to metabolize fructose was previously demonstrated by experiments on cultured brain slices [121,122,123,124]. It was also demonstrated that the cerebellum, the hippocampus, the cortex and other brain regions express GLUT5 and/or 9, as well as the genes of the Fructose-1-Phosphate pathway at levels comparable to those of the liver [125,126], thus actively participating in the fructose metabolism. Several studies also revealed that the fructose metabolism is further enhanced after exposure to high fructose in the diet [125,127,128]. By performing injection of ^14^C-fructose into rat neocortex in vivo or by incubating in vitro isolated neocortical nerve terminals with ^14^C-fructose, it was demonstrated that fructose is taken up into the neocortical cells and nerve terminals that express Hexokinase 1 and genes involved in fructose transport [129]. Further, they showed that fructose detoxification essentially occurs via the oxidative metabolism, involving glycolysis and the tricarboxylic acid cycle. More recently, it was reported that just a one-week fructose diet was associated with an increased expression of GLUT5 in a rat hippocampus [48].

Overall, these investigations strongly suggest that fructose can enter the brain compartment, be metabolized there and exert its direct action on the cells of the CNS. This area of research, although of primary importance due to the demonstrated effects of exogenously administered fructose on brain physiology, has been investigated so far only by a few research groups. In particular, recent reports have evidenced a direct pro-inflammatory action of fructose on glial cells [39,42]. Although the activation of the TLR4/NF-kB pathway is involved in inflammatory response of primary cultured astrocytes to fructose exposure [42], the specific mechanisms by which fructose can directly cause the activation of brain cells and the development of neuroinflammation need further investigation.

Interestingly, in vitro experiments were also carried out using primary cultures of astrocytes or co-cultures involving neurons and astrocytes treated with fructose concentrations ranging from 1mM to 200mM, based on the levels of fructose in systemic circulation after fructose consumption. The results demonstrated that fructose treatment did not promote qualitative changes in the density of astrocytes or affect GLUT5 levels in astrocytic cultures. The levels of this transporter were instead increased in a culture containing both neurons and astrocytes, suggesting that either astrocytes require neuronal interaction or the neurons themselves were the main contributor to the increase in GLUT5 levels [48].

## 8. Conclusions

**Figure 2:**
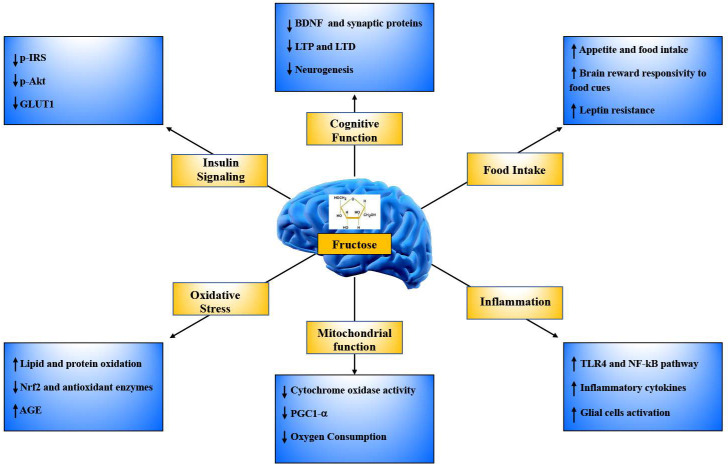
Major brain changes following fructose dietary introduction. The boxes indicate proteins and mechanisms modulated by fructose treatment in the brain in the context of the regulation of food intake, neuroinflammation, mitochondrial function, oxidative stress, insulin signaling and cognitive function. Up arrows indicate parameters stimulated and down arrows indicate parameters reduced by fructose intake. p-IRS = phosphorylated insulin receptor and insulin receptor substrate 1; p-Akt = serine/threonine protein kinase B; GLUT1 = glucose transporter 1; NRF2 = nuclear factor 2; AGE = advanced glycation end products; BDNF = brain derived neurotrophic factor; LTP = long-term potentiation; LTD = long-term depression; PGC1-α = peroxisome proliferator-activated receptor gamma coactivator-1 alpha; TLR4 = toll-like receptor 4; NF-kB = nuclear factor kappa-light-chain-enhancer of activated B cells.

Total fructose consumption has drastically increased during the nineteenth and twentieth centuries due to the widespread use of HFCS as sweeteners for food, beverages, snacks, and baked goods. Fructose overconsumption has long been known to induce overweigh, obesity and inflammation, as well as dyslipidemia, insulin resistance and related metabolic diseases. In the past 10 years, fructose transporters and fructose metabolizing enzymes have been shown to be present in brain cells, such as neurons and glial cells, which were not presumed to metabolize fructose, thus overcoming prevalent dogmas related to brain fructose metabolism. In parallel, it has emerged that fructose consumption, even in the short term, can influence brain function and health by promoting neuroinflammation, an alteration in brain redox balance and insulin signaling (Figure 2). In addition, it can act either on specific areas of the brain involved in the regulation of food intake, motivation and reward mechanisms or on critical regions for learning and memory, influencing synaptic plasticity and cognition, with the hippocampus being one of the most affected (Figure 2). As a result, excess dietary fructose might also promote the onset of mechanisms related to neurodegeneration.

The research area related to the impact of fructose on brain function is certainly less explored than those devoted to the effects of this sugar on other organs. Hence, a challenge for the near future could be to fully elucidate the direct effects of different concentrations of fructose on brain cells, as well as the real amount of this sugar reaching the brain compartment after its dietary ingestion.

The research data collected so far point out that targeted dietary interventions, as well as specific policies, are necessary to reduce the consumption of refined sugars worldwide, not only to prevent overweight and metabolic syndrome but, importantly given the growing aging population, to promote brain health.
